# Prognostic value of the C-reactive protein–albumin–lymphocyte index for predicting one-year mortality in acute pulmonary embolism

**DOI:** 10.3389/fcvm.2026.1928531

**Published:** 2026-10-19

**Authors:** Abdulkadir Kara, Güney Erdoğan, Serkan Sivri, Mustafa Yenerçağ, Faruk Boyacı, Onur Osman Şeker, Ahmet Karagöz, Enes Furkan Öztürk, Süleyman Mert Aşcı

**Affiliations:** 1 Cardiology, Samsun City Hospital, Samsun, Türkiye; 2 Cardiology, Samsun City Hospital, Samsun University, Samsun, Türkiye

**Keywords:** acute pulmonary embolism, CALLY index, inflammation, nutritional status, prognosis

## Abstract

**Background and objectives:**

Acute pulmonary embolism (APE) is associated with substantial mortality despite advances in diagnosis and treatment. Biomarkers reflecting inflammation, immune status, and nutritional reserve may improve risk stratification. This study aimed to evaluate the prognostic significance of the C-reactive protein–albumin–lymphocyte (CALLY) index for predicting one-year mortality in patients with APE and to compare its performance with the Prognostic Nutritional Index (PNI) and Systemic Immune-Inflammation Index (SII).

**Methods:**

This retrospective single-center study included 471 consecutive patients diagnosed with APE between July 2021 and December 2024. The primary endpoint was one-year all-cause mortality. CALLY, PNI, and SII were calculated using admission laboratory parameters. Receiver operating characteristic (ROC) analysis and Cox regression models were performed to evaluate the prognostic performance of these indices.

**Results:**

During one-year follow-up, 102 patients (21.7%) died. CALLY values were significantly lower in non-survivors than in survivors (*p* < 0.001). CALLY demonstrated the highest discriminatory performance for one-year mortality (AUC 0.787, 95% CI 0.732–0.842), compared with PNI (AUC 0.728) and SII (AUC 0.643). CALLY remained independently associated with one-year mortality after multivariable adjustment (HR 0.904, 95% CI 0.856–0.955; *p* < 0.001). Furthermore, adding CALLY to a baseline model including age, sPESI ≥1, elevated cardiac troponin, and sPAP increased the C-statistic from 0.700 to 0.739 and significantly improved model fit (likelihood-ratio *p* < 0.001).

**Conclusions:**

The CALLY index was independently associated with one-year all-cause mortality in patients with APE and demonstrated higher discriminatory performance than PNI and SII. As a simple index derived from routinely available laboratory parameters, CALLY may provide additional prognostic information for long-term risk assessment in APE.

## Introduction

Acute pulmonary embolism (APE) is a life-threatening condition associated with high mortality and morbidity, leading to significant adverse clinical outcomes despite early diagnosis and appropriate treatment (1). Patient prognosis is influenced by numerous clinical factors, including age, the burden of comorbidities, malignancy, right ventricular dysfunction, renal failure, and hemodynamic status (2–4). However, studies conducted in recent years have shown that inflammation, immune responses, and host-specific biological characteristics may also have significant effects on the course of the disease (5, 6). The activation of inflammatory processes may contribute to the development of endothelial dysfunction, increased thrombotic tendency, thrombus persistence, and adverse cardiovascular remodeling processes (7).

The clinical presentation and prognosis of APE vary considerably among patients. While some patients make a full recovery, others may experience rapid clinical deterioration and mortality. Risk assessment in APE patients is primarily based on clinical scoring systems (8–10). In particular, the Pulmonary Embolism Severity Index (PESI) and Simplified PESI (sPESI) are widely used to determine patients’ short-term risk levels (11). These scores enable risk stratification by taking into account age, clinical characteristics, vital signs, and comorbidities. However, current risk classification models provide only limited assessment of the inflammatory and immunological processes that may contribute to disease progression. Importantly, the prognostic burden of APE extends beyond the acute phase, with substantial mortality continuing during long-term follow-up. Long-term outcomes may be influenced not only by the initial severity of PE but also by underlying comorbidities, persistent inflammatory activity, nutritional status, and reduced physiological reserve (12, 13). Therefore, one-year mortality represents a clinically relevant endpoint that may capture broader patient vulnerability beyond short-term risk alone.

As a result, growing attention has been directed toward biomarkers that simultaneously reflect inflammation, immune status, and nutritional condition. Among these, the Prognostic Nutrition Index (PNI), the C-reactive protein-albumin-lymphocyte (CALLY) index, and the Systemic Immune-Inflammation Index (SII) are readily available composite markers that integrate different aspects of inflammation, immune response, and nutritional status. These indices have demonstrated prognostic value across various cardiovascular and inflammatory conditions; however, evidence regarding their roles in APE is limited (14–17). Because these biomarkers may also reflect the patient's underlying biological reserve rather than only the acute thromboembolic event, their prognostic value may extend beyond short-term outcomes. Therefore, the present study aimed to evaluate the prognostic value of the CALLY index for one-year all-cause mortality in patients with APE and to compare its discriminatory performance with PNI and SII.

## Methods

### Study population and study design

This single-center, retrospective, observational study included patients admitted to the Cardiology Clinic at Samsun Training and Research Hospital with a diagnosis of APE between July 2021 and December 2024. The diagnosis of APE was established based on clinical evaluation, laboratory findings, and computed tomography pulmonary angiography (CTPA). Diagnostic and treatment decisions were made according to institutional protocols and routine clinical practice throughout the study period.

A total of 536 patients were screened for eligibility. Patients with unavailable one-year follow-up or mortality data, severe active infections, systemic inflammatory or autoimmune diseases, or advanced liver failure were excluded. Malignancy was an exclusion criterion only in patients with active hematological disease or terminal-stage solid tumors. After applying the exclusion criteria, a total of 471 patients were included in the final analysis set (Figure 1).

**Figure 1:**
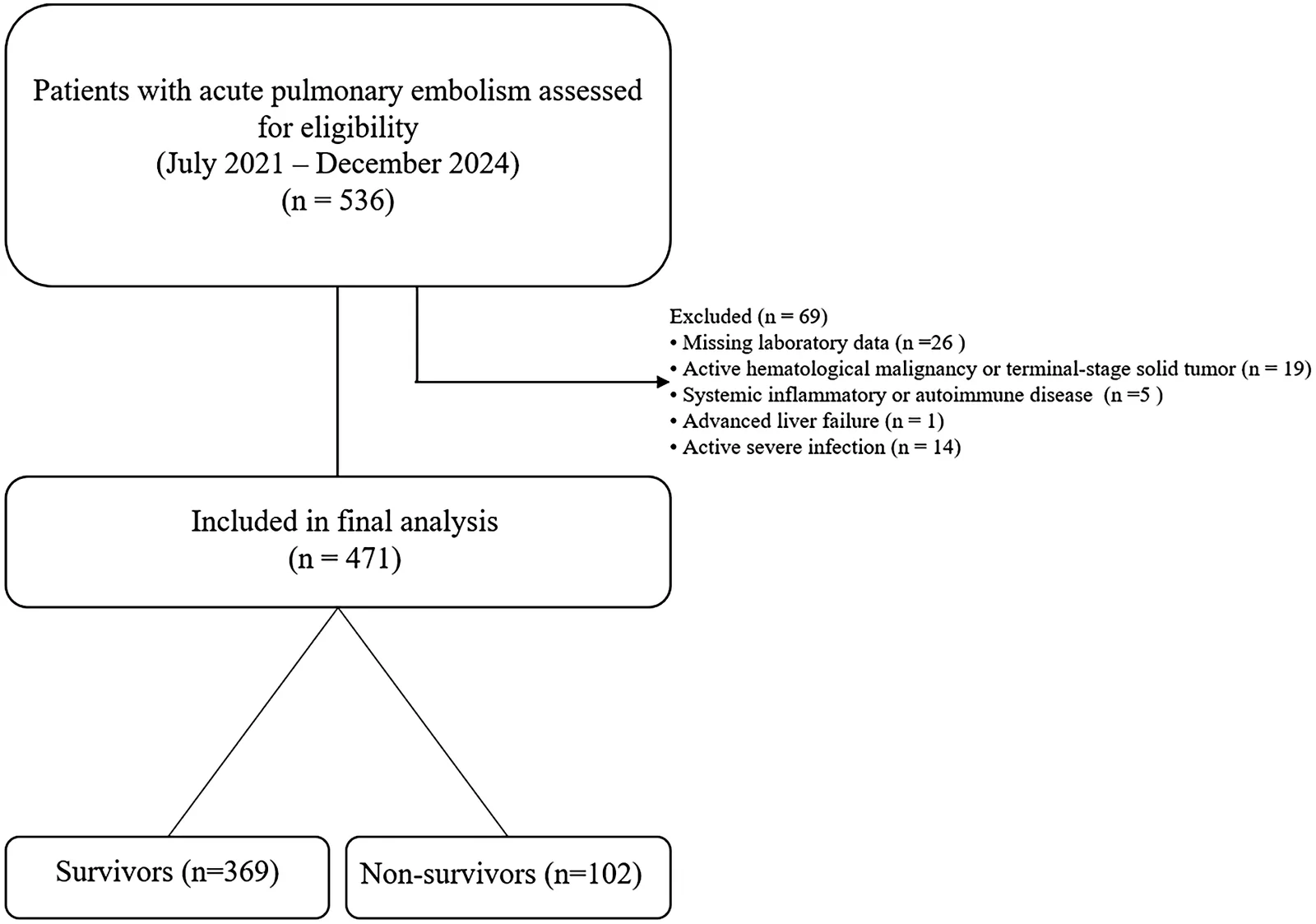
Flow chart of patient selection and study population.

The study was approved by the Samsun University Ethics Committee for Non-Interventional Clinical Research (Protocol No.: GOKAEK 2026/3/24) and was conducted in accordance with the principles of the Declaration of Helsinki.

### Clinical data and risk classification

Demographic data, comorbidities, clinical findings, laboratory parameters, echocardiographic measurements, and follow-up data for the patients were retrospectively obtained from the hospital's electronic medical record system. Information regarding treatment strategies, administration of thrombolytic therapy, and length of hospital stay was also collected.

APE severity was classified according to PE risk stratification criteria as high-risk, intermediate-high-risk, intermediate-low-risk, or low-risk PE. Patients with hemodynamic instability were classified as high-risk PE. In hemodynamically stable patients, further risk stratification was based on the Simplified Pulmonary Embolism Severity Index (sPESI), right ventricular (RV) dysfunction, and cardiac troponin levels. Patients with an sPESI score ≥1 together with both RV dysfunction and elevated cardiac troponin were classified as intermediate-high risk. Hemodynamically stable patients with increased clinical risk and/or RV dysfunction or myocardial injury who did not meet the criteria for intermediate-high risk were classified as intermediate-low risk. Patients with an sPESI score of 0 and without RV dysfunction or elevated cardiac troponin were classified as low-risk PE.

### Laboratory and echocardiographic evaluation

The analyses utilized laboratory data obtained at the time of hospital admission. The parameters evaluated included hemoglobin, hematocrit, white blood cell count, neutrophil, lymphocyte, and platelet counts, CRP, albumin, total protein, renal function tests, D-dimer, and troponin levels. Echocardiographic evaluation was performed using transthoracic echocardiography examinations conducted during the hospital admission process, and left ventricular ejection fraction (LVEF), systolic pulmonary artery pressure (sPAP), right ventricular diameter, and left atrial diameter were recorded.

### Calculation of inflammatory and nutritional indices

To assess inflammation, immune status, and nutritional condition, the PNI, CALLY index, and SII were calculated using laboratory parameters obtained at admission. The PNI was calculated as follows: PNI = 10 × serum albumin (g/dL) + 0.005 × absolute lymphocyte count (/µL). The CALLY index was calculated using the following formula: CALLY = [serum albumin level (g/dL) × absolute lymphocyte count (10^3^/µL)]/[C-reactive protein (mg/dL)]. The SII was calculated as platelet count  ×  neutrophil count/lymphocyte count.

### Endpoints and follow-up

The primary endpoint of the study was defined as one-year all-cause mortality. Mortality data were obtained from hospital records and available follow-up information. Patients for whom one-year mortality data were unavailable were excluded from the study. In addition, 30-day mortality and total length of hospital stay were recorded.

### Statistical analysis

Statistical analyses were performed using IBM SPSS Statistics software (version 26.0, IBM Corp., Armonk, NY, USA). The normality of continuous variables was assessed using the Kolmogorov–Smirnov test. Data showing a normal distribution were presented as mean ± standard deviation, while data not showing a normal distribution were presented as median (interquartile range). Categorical variables were expressed as counts and percentages. For group comparisons, the independent samples t-test was used for normally distributed continuous variables, and the Mann–Whitney U test was applied for non-normally distributed variables. For comparisons of categorical variables, the chi-square test or, when appropriate, Fisher's exact test was applied.

The discriminatory performance of the CALLY, PNI, and SII indices in predicting one-year mortality was evaluated using receiver operating characteristic (ROC) curve analysis, and the area under the curve (AUC) was calculated. Optimal cutoff values were determined using the Youden index. Factors associated with one-year all-cause mortality were initially evaluated using univariable Cox proportional hazards regression analysis. In addition to the cutoff-based analyses, CALLY, PNI, and SII were evaluated as continuous variables in univariable Cox regression analyses. Hazard ratios for CALLY and PNI were expressed per 1-unit increase, whereas the hazard ratio for SII was expressed per 100-unit increase. Variables with *p* < 0.100 in the univariable analysis and parameters considered clinically relevant were considered for multivariable analysis. To avoid potential multicollinearity arising from shared components among the CALLY, PNI, and SII indices, separate multivariable Cox regression models were constructed for each index using the same clinical covariates. Thus, CALLY, PNI, and SII were not entered simultaneously into the same multivariable model. Results were expressed as hazard ratios (HRs) with 95% confidence intervals (CIs). To assess the incremental prognostic value of CALLY beyond established clinical risk predictors, a baseline Cox model including age, sPESI ≥1, elevated cardiac troponin, and systolic pulmonary artery pressure (sPAP) was compared with an extended model additionally including CALLY as a continuous variable. Model discrimination was assessed using the C-statistic, and improvement in model fit after the addition of CALLY was evaluated using the likelihood-ratio test. As a sensitivity analysis, the association between continuous CALLY and one-year mortality was re-evaluated after excluding patients with malignancy and those with missing malignancy status. A parsimonious multivariable Cox model adjusted for age and immobilization was used to account for clinically relevant prognostic factors while avoiding model overfitting. Survival analyses were performed using the Kaplan–Meier method, and differences between groups were assessed using the log-rank test. Kaplan–Meier curves were constructed based on the cutoff values obtained from ROC analyses for CALLY, PNI, and SII. A two-sided *p*-value <0.05 was considered statistically significant.

## Results

A total of 471 patients with APE were included in the study. During the one-year follow-up period, all-cause mortality occurred in 102 patients (21.7%), while 369 patients (78.3%) survived. Patients who died during follow-up were significantly older (69.2 ± 13.3 vs. 63.8 ± 15.7 years, *p* = 0.001) and had a higher prevalence of malignancy (32.4% vs. 12.2%, *p* < 0.001) and immobility (33.3% vs. 14.1%, *p* < 0.001). In addition, the total length of hospital stay was significantly longer in non-survivors than in survivors (10 [7–15] days vs. 9 [5–12] days, *p* = 0.010). The proportion of patients with sPESI ≥1 was significantly higher in the mortality group (*p* < 0.001). PE risk classification differed significantly between survivors and non-survivors (*p* = 0.038), with a lower proportion of low-risk PE among non-survivors (Table 1).

**Table 1:** Baseline demographic and clinical characteristics according to 1-year mortality status.

Parameter	All patients (*n* = 471)	Survivors (*n* = 369)	Non-survivors (*n* = 102)	*p* value
Age (year)	65 ± 15.4	63.8 ± 15.7	69.2 ± 13.3	0.001
Male, (n) %	250 (53.1)	191 (51.8)	59 (57.8)	0.276
Hypertension (n) %	250 (53.1)	194 (52.6)	56 (54.9)	0.677
Diabetes (n) %	111 (23.6)	83 (22.6)	28 (27.5)	0.303
Prior CAD (n) %	117 (24.8)	87 (23.6)	30 (29.7)	0.207
COPD, (n) %	117 (24.8)	88 (23.8)	29 (28.4)	0.343
Prior CVD, (n) %	35 (7.4)	23 (6.3)	12 (11.8)	0.062
AF, (n) %	115 (24.4)	86 (23.3)	29 (28.3)	0.286
Heart Failure, (n) %	80 (17)	61 (16.5)	19 (18.7)	0.618
CKD, (n) %	49 (10.4)	35 (9.5)	14 (13.7)	0.221
Deep Venous Thrombosis, (n) %	161 (34.2)	132 (35.8)	29 (28.4)	0.167
Immobility, (n) %	85 (18)	51 (14.1)	34 (33.3)	0.001
Malignancy, (n) %	77 (16.3)	44 (12.2)	33 (32.4)	0.001
sPESI ≥ 1, (n) %	246 (52.2)	177 (47.9)	69 (67.6)	0.001
PE risk classification, *n* (%)				0.038
Low-risk	91 (19.3)	81 (22.0%)	10 (9.8%)	
Intermediate-low	195 (41.4)	146 (39.6%)	49 (48.0%)	
Intermediate-high	64 (13.6)	47 (12.7%)	17 (16.7%)	
High-risk	121 (25.7)	95 (25.7%)	26 (25.5%)	
Thrombolytic therapy, (n) %	71 (15.1)	61 (13)	10 (9.8)	0.093
Length of stay (days)	9 (5–13)	9 (5–12)	10 (7–15)	0.010

CAD, coronary artery disease; COPD, chronic obstructive pulmonary disease; CVD, cerebrovascular disease; AF, atrial fibrillation; CKD, chronic kidney disease; DVT, deep vein thrombosis; LoS, length of stay;sPESI, simplified Pulmonary Embolism Severity Index.

Laboratory evaluations revealed more pronounced inflammatory activity and poorer nutritional profiles in patients who died. CRP, neutrophil count, white blood cell count, D-dimer levels, and troponin levels were significantly higher in the mortality group, while lymphocyte count, hemoglobin, hematocrit, albumin, and total protein levels were significantly lower (all *p* < 0.05). In contrast, creatinine, estimated glomerular filtration rate, platelet count, AST, and ALT levels did not differ significantly between groups (Table 2).

**Table 2:** Laboratory and echocardiographic parameters according to 1-year mortality status.

Parameter	All patients (*n* = 471)	Survivors (*n* = 369)	Non-survivors (*n* = 102)	*p* value
Laboratory parameters				
Hemoglobin (g/dL)	12.3 ± 2.2	12.5 ± 2.1	11.7 ± 2.3	0.001
Hematocrit, %	37.3 ± 6.1	37.8 ± 5.9	35.6 ± 6.6	0.001
Creatinine (mg/dL)	0.9 ± 0.4	0.9 ± 0.3	0.9 ± 0.5	0.978
eGFR, mL/min/1.73 m^2^	72.6 ± 12.9	72.7 ± 13.2	72.3 ± 11.8	0.786
Urea (mg/dL)	41.7 (31–57)	41 (30–56)	44.8 (33–66)	0.069
Triglyceride, mg/dL	143 (105–188)	144 (110–188)	130 (91–187)	0.256
LDL-Cholesterol, mg/dL	120.3 ± 36.5	121.1 ± 37.2	117.2 ± 33.7	0.128
HDL-Cholesterol, mg/dL	45.7 ± 14.8	46.1 ± 15.1	44.2 ± 13.7	0.096
WBC (×1,000/µL)	8.8 (6.3–10.7)	8.1 (6.1–10.4)	8.8 (6.9–12.1)	0.031
Lymphocyte (×1,000/µL)	1.6 ± 0.8	1.7 ± 0.8	1.4 ± 0.8	0.001
Neutrophil (×1,000/µL)	6.2 ± 3.3	5.9 ± 3.2	7.1 ± 3.7	0.001
Monocyte (×1,000/µL)	0.6 ± 0.2	0.6 ± 0.2	0.6 ± 0.3	0.509
Platelet count (×1,000/µL)	257.1 ± 96.3	259 ± 97	250 ± 94	0.399
AST (U/L)	26 (19–37)	26 (19–37)	23.6 (16–36)	0.155
ALT (U/L)	22.7 (14–36)	24 (14–37)	19 (11–30)	0.134
CRP (mg/dL)	3.58 ± 4.02	2.93 ± 3.33	5.94 ± 5.44	0.001
Total protein (g/L)	63.3 ± 8.7	64.1 ± 8.8	60.5 ± 7.7	0.001
Albumin (g/dL)	3.5 ± 0.5	3.6 ± 0.5	3.2 ± 0.5	0.001
D-dimer (mg/L)	3.8 (1.8–8.8)	3.5 (1.6–8.6)	6.4 (2.9–9.8)	0.001
Elevated troponin, *n* (%)	133 (28.2)	78 (21.1)	55 (53.9)	<0.001
PNI	44 (39.2–49)	45 (40.5–50)	39.5 (34.6–43.6)	<0.001
SII	880 (533–1,514)	737 (483–1,047)	1,373 (816–2,411)	<0.001
CALLY index	2.3 (0.8–7.4)	3.4 (1.3–8.2)	0.5 (0.3–1.7)	<0.001
Echocardiographic parameters				
LVEF (%)	55.0 ± 8.1	55.3 ± 8.1	54.1 ± 8.1	0.204
sPAP (mmHg)	37.9 ± 13.8	37.1 ± 13.9	40.8 ± 13	0.011
RV Diameter (mm)	32.7 ± 4.4	32.4 ± 4.5	33.8 ± 4	0.004
LA Diameter (mm)	35.7 ± 4.3	35.1 ± 4.1	37.7 ± 4.6	0.001

eGFR, estimated glomerular filtration rate; HDL-C, high-density lipoprotein cholesterol; LDL-C, low-density lipoprotein cholesterol; WBC, white blood cell count; AST, aspartate aminotransferase; ALT, alanine aminotransferase; CRP, C-reactive protein; PNI, prognostic nutritional index; SII, systemic immune-inflammation index; CALLY, C-reactive protein–albumin–lymphocyte index; LVEF, left ventricular ejection fraction; sPAP, systolic pulmonary artery pressure; RV, right ventricular; LA, left atrial.

Echocardiographic evaluation revealed more pronounced right ventricular overload in patients who died. Systolic pulmonary artery pressure (40.8 ± 13.0 vs. 37.1 ± 13.9 mmHg, *p* = 0.011), right ventricular diameter (33.8 ± 4.0 vs. 32.4 ± 4.5 mm, *p* = 0.004), and left atrial diameter (37.7 ± 4.6 vs. 35.1 ± 4.1 mm, *p* = 0.001) were significantly higher in non-survivors. By contrast, no significant difference was observed in left ventricular ejection fraction (54.1 ± 8.1 vs. 55.3 ± 8.1, *p* = 0.204) (Table 2).

Consistent with the laboratory findings, PNI and CALLY values were significantly lower, whereas SII values were significantly higher, in patients who died during follow-up. Median PNI was significantly lower in non-survivors than in survivors (39.5 [34.6–43.6] vs. 45 [40.5–50], *p* < 0.001). Similarly, the median CALLY index was significantly lower in non-survivors (0.5 [0.3–1.7] vs. 3.4 [1.3–8.2], *p* < 0.001), whereas median SII was significantly higher (1,373 [816–2,411] vs. 737 [483–1,047], *p* < 0.001) (Table 2).

In the ROC curve analysis (Figure 2), the CALLY index was shown to have the highest discriminatory performance in predicting one-year mortality (AUC: 0.787; 95% CI: 0.732–0.842; *p* < 0.001). The AUC value for PNI was 0.728 (95% CI: 0.672–0.784; *p* < 0.001), and for SII, it was 0.643 (95% CI: 0.580–0.706; *p* < 0.001). Optimal cutoff values were ≤1.6 for CALLY, ≤42 for PNI and ≥996 for SII (Figure 2). Pairwise comparison of ROC curves using the DeLong test demonstrated that the AUC of the CALLY index was significantly higher than that of both PNI (*p* = 0.034) and SII (*p* < 0.001).

**Figure 2:**
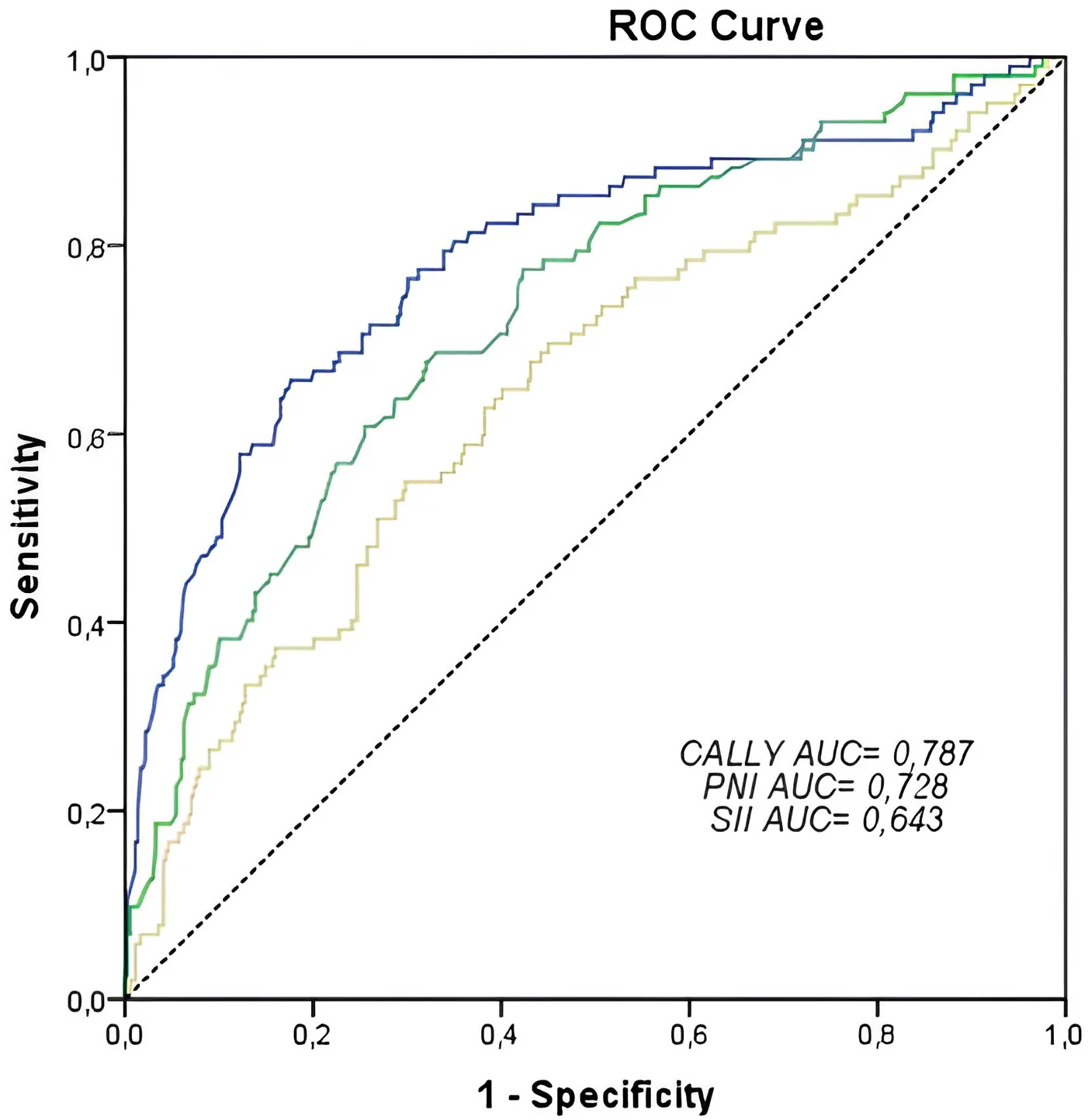
ROC curve analysis of CALLY, PNI, and SII for the prediction of one-year mortality.

In analyses based on the defined cutoff values, significantly worse clinical outcomes were observed in the low CALLY group. The one-year mortality rate was 40.8% in patients with CALLY ≤1.6, whereas it was 9.9% in patients with CALLY >1.6 (*p* = 0.001). Similarly, the 30-day mortality rate was significantly higher in the low CALLY group (14.5% vs. 3.8%, *p* = 0.001). No significant difference was observed between the groups in terms of total length of hospital stay (9.5 [5–14] days vs. 9 [5–12] days, *p* = 0.241). Additionally, patients with CALLY ≤1.6 more frequently had an sPESI score ≥1 compared with those with CALLY >1.6 (58.7% vs. 48.3%, *p* = 0.028). The distribution of PE risk categories differed significantly between the CALLY groups (*p* = 0.007). High-risk PE was more frequent in patients with CALLY ≤1.6 than in those with CALLY >1.6 (32.4% vs. 21.6%), whereas low-risk PE was less frequent (13.4% vs. 22.9%) (Table 3).

**Table 3:** Clinical outcomes and risk stratification according to CALLY index groups.

Parameter	CALLY >1.6 (*n* = 292)	CALLY ≤1.6 (*n* = 179)	*p* value
1 year Mortality, n(%)	29 (9.9)	73 (40.8)	0.001
30 days Mortality, n(%)	11 (3.8)	26 (14.5)	0.001
Total Length of Stay (days)	9 (5–12)	9.5 (5–14)	0.241
PE risk classification, *n* (%)			0.007
Low-risk	67 (22.9)	24 (13.4)	
Intermediate-low	126 (43.2)	68 (38)	
Intermediate-high	36 (12.3)	29 (16.2)	
High-risk	63 (21.6)	58 (32.4)	
sPESI ≥1, n(%)	141 (48.3)	105 (58.7)	0.028

sPESI, Simplified Pulmonary Embolism Severity Index.

In univariable Cox regression analysis, age, malignancy, immobilization, sPAP, and elevated troponin were significantly associated with one-year mortality. When analyzed as continuous variables, CALLY (HR 0.895, 95% CI 0.847–0.946; *p* < 0.001), PNI (HR 0.902, 95% CI 0.877–0.929; *p* < 0.001), and SII (HR 1.028 per 100-unit increase, 95% CI 1.017–1.040; *p* < 0.001) were also significantly associated with one-year mortality.

To avoid potential multicollinearity among the inflammatory and nutritional indices, separate multivariable Cox regression models were constructed for CALLY, PNI, and SII, each adjusted for age, sPESI ≥1, elevated cardiac troponin, and sPAP. In these models, higher CALLY (HR 0.904, 95% CI 0.856–0.955; *p* < 0.001) and PNI (HR 0.911, 95% CI 0.885–0.938; *p* < 0.001) were independently associated with lower one-year mortality, whereas higher SII was independently associated with increased mortality (HR 1.020 per 100-unit increase, 95% CI 1.009–1.032; *p* = 0.001) (Table 4).

**Table 4:** Univariable and multivariable cox regression analyses for predictors of 1-year mortality in patients with acute pulmonary embolism.

Analysis		Univariable				Multivariable^a^		
Parameter	Hazard Ratio (HR)	95% CI for HR		P	Hazard Ratio (HR)	95% CI for HR		P
		Lower	Upper			Lower	Upper	
Age	1.022	1.008–1.037		0.002	1.007	0.993–1.022		0.333
Male sex	1.253	0.846–1.856		0.261				
Hypertension	1.098	0.743–1.622		0.638				
Diabetes mellitus	1.298	0.840–2.005		0.240				
eGFR (mL/min/1.73 m^2^)	0.998	0.983–1.013		0.773				
History of CAD	1.388	0.906–2.127		0.132				
History of Stroke/TIA	1.734	0.950–3.168		0.073				
History of Heart Failure	1.173	0.712–1.930		0.531				
Previous Atrial Fibrillation	1.294	0.841–1.989		0.241				
COPD	1.232	0.802–1.895		0.341				
Malignancy	2.885	1.905–4.371		0.001				
Immobility	2.480	1.643–3.745		0.001				
sPESI≥1	2.670	1.728–4.125		<0.001	1.683	1.061–2.668		0.027
LVEF, %	0.991	0.969–1.014		0.447				
sPAP, mmHg	1.017	1.004–1.031		0.012	1.011	0.997–1.026		0.118
Elevated Troponin	3.687	2.497–5.445		<0.001	2.943	1.976–4.383		<0.001
PNI	0.902	0.877–0.929		<0.001				
SII	1.028	1.017–1.040		<0.001				
CALLY	0.895	0.847–0.946		<0.001	0.904	0.856–0.955		<0.001

HR, hazard ratio; CI, confidence interval; CAD, coronary artery disease; TIA, transient ischemic attack; eGFR, estimated glomerular filtration rate; COPD, chronic obstructive pulmonary disease; LVEF, left ventricular ejection fraction; sPESI, simplified Pulmonary Embolism Severity Index; sPAP, systolic pulmonary artery pressure; PNI, prognostic nutritional index; SII, systemic immune-inflammation index; CALLY, C-reactive protein–albumin–lymphocyte index.

a Multivariable estimates presented in the table correspond to the CALLY-specific model. Separate multivariable models were constructed for PNI and SII to avoid potential multicollinearity among the indices.

To assess the incremental prognostic value of CALLY, a baseline model including age, sPESI ≥1, elevated cardiac troponin, and sPAP was compared with an extended model additionally including continuous CALLY. The C-statistic increased from 0.700 to 0.739 after the addition of CALLY (*Δ*C-statistic=0.040), and the likelihood-ratio test demonstrated a significant improvement in model fit (*χ*^2^ = 25.03; *p* < 0.001).

In the sensitivity analysis restricted to 385 patients without malignancy, CALLY remained significantly associated with one-year mortality when analyzed as a continuous variable (HR 0.898, 95% CI 0.845–0.954; *p* < 0.001). This association remained significant after adjustment for age and immobilization (adjusted HR 0.899, 95% CI 0.846–0.956; *p* < 0.001).

Finally, in the Kaplan–Meier survival analysis, one-year survival was shown to be significantly lower in patients with CALLY ≤1.6, PNI ≤42, and SII ≥996 (log-rank *p* < 0.001 for all analyses) (Figure 3).

**Figure 3:**
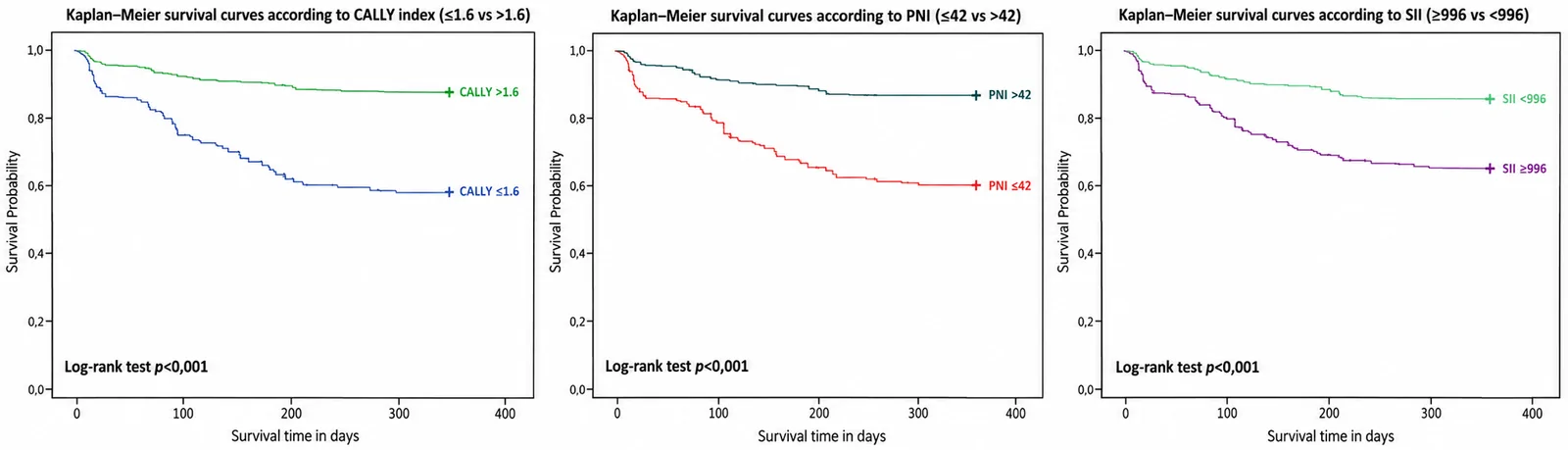
Kaplan–meier survival curves according to CALLY, PNI, and SII groups.

## Discussion

Our study yielded several important findings: (1) CALLY, PNI, and SII were significantly associated with one-year mortality in patients with APE; (2) among the evaluated indices, CALLY demonstrated the highest discriminatory performance; (3) when evaluated in separate multivariable models to avoid potential multicollinearity, CALLY, PNI, and SII each remained independently associated with one-year mortality; and (4) the addition of continuous CALLY to a baseline model including established clinical risk parameters improved model discrimination and model fit. Together, these findings suggest that inflammation- and nutrition-based indices, particularly CALLY, may provide additional prognostic information in patients with APE.

The CALLY index is a composite inflammatory-nutritional marker derived from CRP, serum albumin, and lymphocyte count, enabling the simultaneous assessment of systemic inflammation, immune status, and nutritional reserves. Although it was initially developed in patients with hepatocellular carcinoma, growing evidence has highlighted its prognostic value across a range of cardiovascular disorders (18). Most notably, low CALLY levels have been identified as independent predictors of poor prognosis in heart failure, acute coronary syndromes, and structural heart diseases (19–21). Similarly, Han et al. reported an association between lower CALLY values and increased all-cause and cardiovascular mortality in patients with cardiovascular disease (22). Moreover, in patients with ST-segment elevation myocardial infarction, Demir et al. showed that reduced CALLY levels were associated with higher in-hospital mortality (23), while Arın et al. demonstrated that a low CALLY index is associated with an increased risk of mortality in patients with heart failure (24).

Our findings extend these observations to patients with APE. Notably, both early and long-term mortality were significantly higher in the low CALLY group. The one-year mortality rate in patients with a CALLY score of ≤1.6 was significantly higher than in the high CALLY group (40.8% vs. 9.9%). Similarly, 30-day mortality was also higher in the low CALLY group (14.5% vs. 3.8%). In contrast, no significant difference was observed between the groups in terms of total length of hospital stay. This suggests that the CALLY index may primarily reflect the patient's underlying biological reserve and long-term vulnerability rather than factors influencing the duration of hospitalization. Furthermore, the association of low CALLY values with both early and late-stage mortality supports its potential role as a clinically useful tool for risk stratification in APE. Importantly, the association between CALLY and one-year mortality remained significant when CALLY was analyzed as a continuous variable in the sensitivity analysis restricted to patients without malignancy. This finding suggests that the prognostic association of CALLY was not solely driven by the higher prevalence of malignancy among non-survivors. Furthermore, CALLY provided incremental prognostic information beyond established clinical risk parameters. The addition of continuous CALLY to a baseline model including age, sPESI ≥1, elevated cardiac troponin, and sPAP increased the C-statistic from 0.700 to 0.739 and significantly improved model fit. These findings suggest that CALLY may capture prognostic information related to inflammatory, immune, and nutritional status that is not fully represented by conventional clinical risk assessment.

The superior performance of the CALLY index may be explained by the complementary biological information provided by its individual components—CRP, lymphocyte count, albumin. First, CRP is a key marker of the acute-phase response and reflects heightened inflammatory activity, endothelial dysfunction, and increased thrombotic burden (25). Synthesized and released by hepatocytes under the upstream regulatory influence of proinflammatory cytokines such as interleukin-1 (IL-1) and interleukin-6 (IL-6), CRP is among the most sensitive and widely used biomarkers of inflammation (26). Owing to its standardized measurement, relatively long half-life, and well-established prognostic value in cardiovascular disease, it has become an important component of cardiovascular risk assessment (27). Additionally, in the APE context, Najarro et al. demonstrated that elevated CRP levels are associated with right ventricular dysfunction and increased mortality risk (28).

The second component, lymphocytes, play a significant role in regulating the inflammatory response and maintaining immune homeostasis. Low lymphocyte levels are associated with increased inflammatory burden, impaired immune function, and greater systemic stress, and may reflect poor clinical outcomes. In contrast, it has been reported that optimal lymphocyte levels may be associated with a stronger immune response and a better clinical condition. Furthermore, a meta-analysis conducted by Li et al. demonstrated that the lymphocyte-based immune-inflammatory response holds prognostic significance in patients with pulmonary embolism (29).

Albumin, the third component of the CALLY index, is not merely an indicator of nutritional status; it also possesses anti-inflammatory, antioxidant, and antithrombotic properties. Low albumin levels may reflect systemic inflammation, a catabolic state, hypercoagulability, and impaired physiological reserve (30). Studies in patients with pulmonary embolism have reported that low serum albumin levels are associated with greater disease severity and a poor short-term prognosis (31, 32).

Taken together, the integration of these three components—CRP, lymphocyte count, and albumin—into a single index may provide a more comprehensive assessment of the biological processes underlying disease progression than any individual marker alone. This concept is particularly relevant to the pathophysiology of APE, where inflammatory activation following a thromboembolic event, right ventricular overload, endothelial damage, and ongoing thrombotic processes may play a role in disease progression. Additionally, concomitant malnutrition, reduced physiological reserve, and immune dysfunction may negatively impact long-term outcomes, especially among older patients and those with multiple comorbidities (33, 34). Supporting this hypothesis, patients who died during follow-up in our cohort exhibited higher CRP levels together with lower albumin and lymphocyte counts. Thus, the CALLY index appears to capture not only inflammatory burden but also the broader biological vulnerability of the patient, which may explain its superior prognostic performance in our study population.

In addition to CALLY, the PNI was identified as an independent predictor of one-year mortality. As a marker integrating nutritional status and immune competence, PNI has previously been associated with adverse outcomes across a variety of cardiovascular conditions (35–37). Furthermore, several studies have reported an association between low PNI values and unfavorable short- and long-term outcomes in patients with APE (38, 39). Our findings extend this evidence by demonstrating that reduced PNI remains independently associated with long-term mortality, which is further supported by the observation of significantly lower albumin and lymphocyte levels, particularly in patients who died.

Meanwhile, SII was also independently associated with one-year mortality when evaluated in a separate multivariable model. This finding supports the prognostic relevance of systemic inflammatory and immune activation in APE. However, SII demonstrated lower discriminatory performance than CALLY in ROC analysis, suggesting that inflammation alone may not fully capture the complex biological processes associated with long-term prognosis. The pathophysiology of APE involves not only inflammatory activation but also interactions among immune responses, thrombotic burden, nutritional status, and host-specific biological characteristics (40). The integration of these different biological domains may partly explain the superior discriminatory performance observed with the CALLY index in our study.

Another important finding was the presence of a more pronounced inflammatory burden and a poorer nutritional profile in patients who died during follow-up. In these patients, CRP, neutrophil count, and troponin positivity were higher, while albumin, total protein, and lymphocyte levels were lower. Moreover, systolic pulmonary artery pressure and right ventricular diameter were significantly higher among non-survivors. These findings suggest that inflammation, right ventricular overload, and impaired physiological reserve may coexist in patients with a poor prognosis.

Despite the insights it offers, it should also be acknowledged that our study has several limitations. First, its retrospective and single-center design limit the generalizability of our findings. Second, inflammatory indices were calculated using only parameters at the time of presentation; therefore, the prognostic significance of temporal changes during follow-up could not be evaluated. Third, the primary endpoint was all-cause mortality, while cause-specific mortality related to pulmonary embolism was not available. In addition, unmeasured differences in clinical management may have contributed to residual confounding. Fourth, despite multivariable adjustment, residual confounding cannot be completely excluded because of the observational nature of the study. Finally, the CALLY cutoff value of 1.6 was derived from the present cohort using the Youden index and was subsequently evaluated within the same population, which may have resulted in an optimistic estimate of its prognostic performance. Therefore, this threshold should be considered exploratory and requires validation in an independent external cohort before clinical application.

In conclusion, our study showed that inflammation- and nutrition-based indices are significantly associated with long-term prognosis in patients with APE. Among the evaluated indices, the CALLY index demonstrated the highest discriminatory performance and remained independently associated with one-year mortality. These findings suggest that the combined assessment of inflammation and nutritional status may contribute to long-term risk stratification in APE.

## Data Availability

The raw data supporting the conclusions of this article will be made available by the authors, without undue reservation.
